# Treatment and Attrition Trends for Metastatic Clear Cell Renal Cell Carcinoma in the US

**DOI:** 10.1001/jamanetworkopen.2025.1201

**Published:** 2025-03-20

**Authors:** Zeynep Irem Ozay, Yeonjung Jo, Gliceida Galarza Fortuna, Chadi Hage Chehade, Georges Gebrael, Micah Ostrowski, Nicolas Sayegh, Ethan Anderson, Salvador Jaime-Casas, Miguel Zugman, Vinay Mathew Thomas, Benjamin L. Maughan, Neeraj Agarwal, Sumanta K. Pal, Umang Swami

**Affiliations:** 1 Division of Medical Oncology, Department of Internal Medicine, Huntsman Cancer Institute, University of Utah, Salt Lake City; 2 Division of Biostatistics, Department of Population Health Sciences, School of Medicine, University of Utah, Salt Lake City; 3 Cancer Biostatistics, Huntsman Cancer Institute, University of Utah, Salt Lake City; 4 Department of Internal Medicine, University of Texas Southwestern Medical Center, Dallas; 5 Division of Medical Oncology, City of Hope Cancer Center, Duarte, California

## Abstract

**Importance:**

The treatment landscape of metastatic clear cell renal cell carcinoma (ccRCC) has rapidly evolved with the approval of multiple immune checkpoint inhibitor (ICI)-based combinations. However, clinical data on changes in treatment patterns and attrition before and after ICI-based combinations approval are lacking.

**Objective:**

To assess treatment patterns and attrition rates in patients with metastatic ccRCC before and after the approval of ICI-based combinations.

**Design, Setting, and Participants:**

This cohort study used patient-level data from a nationwide deidentified electronic health record-derived database, originating from around 280 cancer clinics in the US. Patients diagnosed with metastatic ccRCC who received first-line therapy between January 1, 2011, and January 20, 2023, were included. Those treated for 2 or more malignant neoplasms or enrolled in clinical trials were excluded.

**Exposures:**

Line of therapy initiation before and after April 16, 2018.

**Main Outcomes Measures:**

Treatments received in each line of therapy and attrition rate were summarized using frequencies and percentages.

**Results:**

Of 12 707 patients with metastatic ccRCC within the database, 8534 were eligible and included (median [IQR] age, 66 [59-74] years; 6032 male [70.7%]; 629 Black [8.1%], 697 Hispanic [9.0%], 5493 White [71.0%]). Before April 16, 2018, the most common first-line therapy was tyrosine kinase inhibitor (TKI) monotherapy (3595 of 4561 patients [78.8%]). Following the approval of ICI-based combinations in 2018, most patients (2392 of 3973 patients [60.2%]) received ICI-based combinations as first-line therapy for metastatic ccRCC. TKI monotherapy remained the most common second- and third-line therapy in patients treated before and after April 16, 2018. Before 2018, 2639 patients (57.9%) and 1458 patients (31.9%) received second-line and third-line therapies, respectively, compared with 1494 (37.6%) and 562 (14.1%) after 2018.

**Conclusions and Relevance:**

In this cohort study of 8534 patients with metastatic ccRCC, although ICI-based combinations are the preferred first-line therapy due to their proven superiority over TKI monotherapy, many patients were not receiving them; high attrition rates were observed in subsequent lines. These findings highlight the need to optimize treatment selection by implementing current guidelines in clinical practice.

## Key Points

**Question:**

What are current treatment patterns and attrition rates in metastatic clear cell renal cell carcinoma (ccRCC)?

**Findings:**

In this cohort study of 8534 patients with metastatic ccRCC, the most common first-line therapy before the approval of immune-checkpoint inhibitor (ICI)-based combinations was tyrosine kinase inhibitor monotherapy (78.8%); following their approval, ICI-based combinations were the most common first-line therapy (60.2%). Only 48.4% and 23.7% of patients received second- and third-line therapies, respectively.

**Meaning:**

The low use of ICI-based combinations in the first line and high attrition rates in subsequent therapy demonstrate the need to improve first-line treatment selection and expand therapeutic options for metastatic ccRCC.

## Teaser

This cohort study of US adults with metastatic clear cell renal cell carcinoma examines changes in forms of treatment used following the approval of immune-checkpoint inhibitor combinations by the US Food and Drug Administration.

## Introduction

Renal cell carcinoma (RCC) is the most common form of kidney cancer and ranks among the 10 most frequent cancers in the US.^1^ In 2024, around 81 610 new cases of RCC were expected, accounting for 4.1% of all new cancer diagnoses and 2.4% of all cancer-associated deaths.^2^ Although most cases are diagnosed in the early stage, around 15% will present with metastatic cancer, which remains a lethal disease.^2^ Clear cell is the most common histology in RCC, accounting for 75% to 85% of all RCC cases.^3,4^

The treatment landscape of metastatic clear cell RCC (ccRCC) has seen unprecedented advances in recent years. Following the cytokine era of interleukin-2 and interferon-alfa,^5,6^ sunitinib became the first vascular endothelial growth factor targeting tyrosine kinase inhibitor (TKI) to garner regulatory Food and Drug Administration (FDA) approval after significantly improving survival outcomes of patients with metastatic ccRCC.^7^ After that, multiple TKIs demonstrated improved survival outcomes in metastatic ccRCC and received FDA approval.^8,9,10^ More recently, the standard of care for first-line management of patients with metastatic ccRCC has seen a paradigm shift toward immune checkpoint inhibitor (ICI)-based combinations. These include dual ICI combinations (ie, ipilimumab with nivolumab) or the combinations of ICI with TKIs (ie, nivolumab with cabozantinib, pembrolizumab with lenvatinib, pembrolizumab with axitinib, and avelumab with axitinib).^11,12,13,14,15^ These regimens have dramatically improved overall survival (OS), reaching a median of around 53 months in the CheckMate 214 trial with the combination of ipilimumab and nivolumab, with 42.8% of patients estimated to be still alive at 90 months.^16^

After progression on ICI-based combinations, there is limited data to guide treatment selection in the second-line setting. Cabozantinib is a multi-TKI, which was approved based on the results of the METEOR trial^8^; however, patients included in this trial had progressed on prior TKIs and not on current standard-of-care ICI-based regimens. Furthermore, understanding the biology of RCC and its interaction with the hypoxia-inducible factor (HIF) pathway has introduced the therapeutic option of belzutifan, a HIF-2α inhibitor.^17^

Given the plethora of treatment options for managing patients with metastatic ccRCC and the lack of clinical data, we sought to assess the current treatment patterns and attrition rates in patients with metastatic ccRCC in a large nationwide study. While a previous clinical study has demonstrated increased use of ICI-based combinations in community practice since their approval,^18^ there remains limited information on detailed treatment trends and attrition rates. This study aims to address these gaps by evaluating treatment patterns and attrition rates in a large nationwide cohort of patients with metastatic ccRCC.

## Methods

### Study Design

This retrospective cohort utilized the Flatiron Health Electronic Health Record (EHR)-derived deidentified database.^19^ This nationwide longitudinal database comprises deidentified patient-level structured and unstructured data, curated through technology-enabled abstraction from around 280 US cancer clinics (approximately 800 sites of care), primarily in community oncology settings.^20,21^ The deidentification process for this purpose is performed in accordance with the Health Insurance Portability and Accountability Act (HIPAA).^21^ This cohort study was approved by the institutional review board at the University of Utah, and informed consent was waived due to the utilization of deidentified data. The study followed the Strengthening the Reporting of Observational Studies in Epidemiology (STROBE) guidelines for reporting cohort studies.

### Patient Population

The study included patients diagnosed with metastatic RCC with clear-cell histology between January 1, 2011, and January 20, 2023, and had first-line treatment information available. Patients receiving systemic therapy (nonhormonal) for 2 or more malignant neoplasms or enrolled in clinical trials were excluded. They initiated first-line treatment from December 18, 2010, through January 31, 2023; second-line treatment from April 1, 2011, through January 31, 2023; and third-line treatment from September 19, 2011, through January 31, 2023. Patients were divided into 2 cohorts based on the date they initiated first-line treatment: cohort 1 included patients who initiated first-line treatment before April 16, 2018, and cohort 2 included those receiving first-line treatment after this date. The date of April 16, 2018, marking the FDA approval of the first ICI-based combination (ipilimumab plus nivolumab) for treating metastatic ccRCC, was chosen as a reference point to divide patients into cohorts for comparing treatment trends before and after this milestone.^22^ For the attrition rate analysis at 1 year and 6 months after treatments, the patient population was restricted to those who ended or discontinued treatment 1 year (January 31, 2022) or 6 months (July 31, 2022) before the last follow-up date in the data, January 31, 2023. Baseline characteristics, including age, gender, and race and/or ethnicity, were collected. Race and ethnicity data were obtained from deidentified HER (including Asian, Black, Hispanic, White, and other [Alaska Native, American Indian, Native Hawaiian or Pacific Islander, or multiracial]), where clinical teams registered this information. These data are usually self-reported by patients through intake interviews and forms, with some variations among practices.

### Outcomes

The study’s primary outcome was to evaluate the trends in treatment patterns of patients with metastatic ccRCC. Attrition rates in the overall cohort and at 1 year and 6 months in cohorts 1 and 2 were calculated to account for treatment-free intervals with ICI-based combinations.

### Statistical Analysis

In the treatment landscape analysis, types of therapy regimens received in the first-line to third-line settings were summarized using frequency and percentages. Attrition rates at 1 year and 6 months were also summarized accordingly. Patients were categorized based on their status at 1 year or 6 months after discontinuing their treatment. Patients who had any record of subsequent treatment received within 1 year or 6 months of discontinuing their prior therapy were categorized as having received subsequent treatment; patients with recorded death without receiving subsequent therapy within a year or 6 months of their last treatment discontinuation were classified as died without subsequent therapy and patients who were lost to follow-up or remained alive without receiving further treatment within 1 year or 6 months of discontinuing their last therapy were categorized as discontinued therapy or remained alive. For attrition rate analysis at 1 year and 6 months after treatment, only patients who had discontinued treatment 1 year or 6 months before the last follow-up date in the data, January 31, 2023, were included. All the analysis was done using R version 4.2.3 (R Project for Statistical Computing).

## Results

Among 12 707 patients with metastatic ccRCC in the database, 8534 were eligible and included in the overall cohort for analysis. The median (IQR) age was 66 (59-74) years; 6032 patients were male (70.7%); 629 patients identified as Black (8.1%), 697 as Hispanic (9.0%), and 5493 as White (71.0%). Baseline characteristics are summarized in eTable 1 in Supplement 1, and the study flow diagram is presented in eFigure 1 in Supplement 1.

In the overall cohort, 4133 patients (48.4%) received second-line therapy, and 2020 (23.7%) received third-line therapy. The most frequently used treatment across all lines of therapy was TKI monotherapy (first-line, 4526 patients [53.0%]; second-line, 1688 patients [40.8%]; third-line, 974 patients [48.2%]).

**Figure 1.**
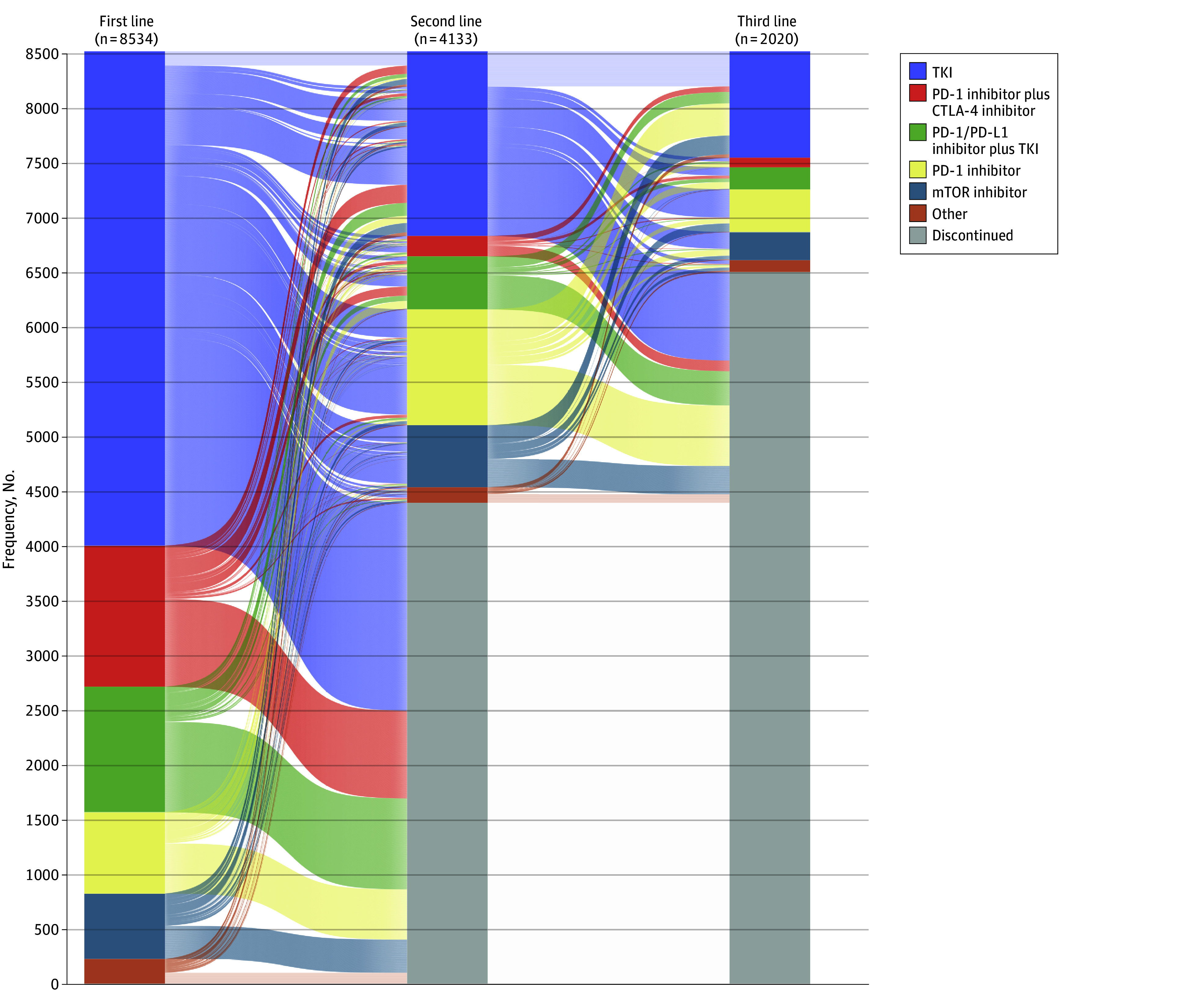
Treatment Trends in the Overall Cohort Across Lines of Therapy CTLA-4 indicates cytotoxic T-lymphocyte–associated antigen 4; mTOR, mammalian target of rapamycin; PD-1, programmed cell death protein 1; PD-L1, programmed cell death ligand 1; TKI, tyrosine kinase inhibitor.

**Figure 2.**
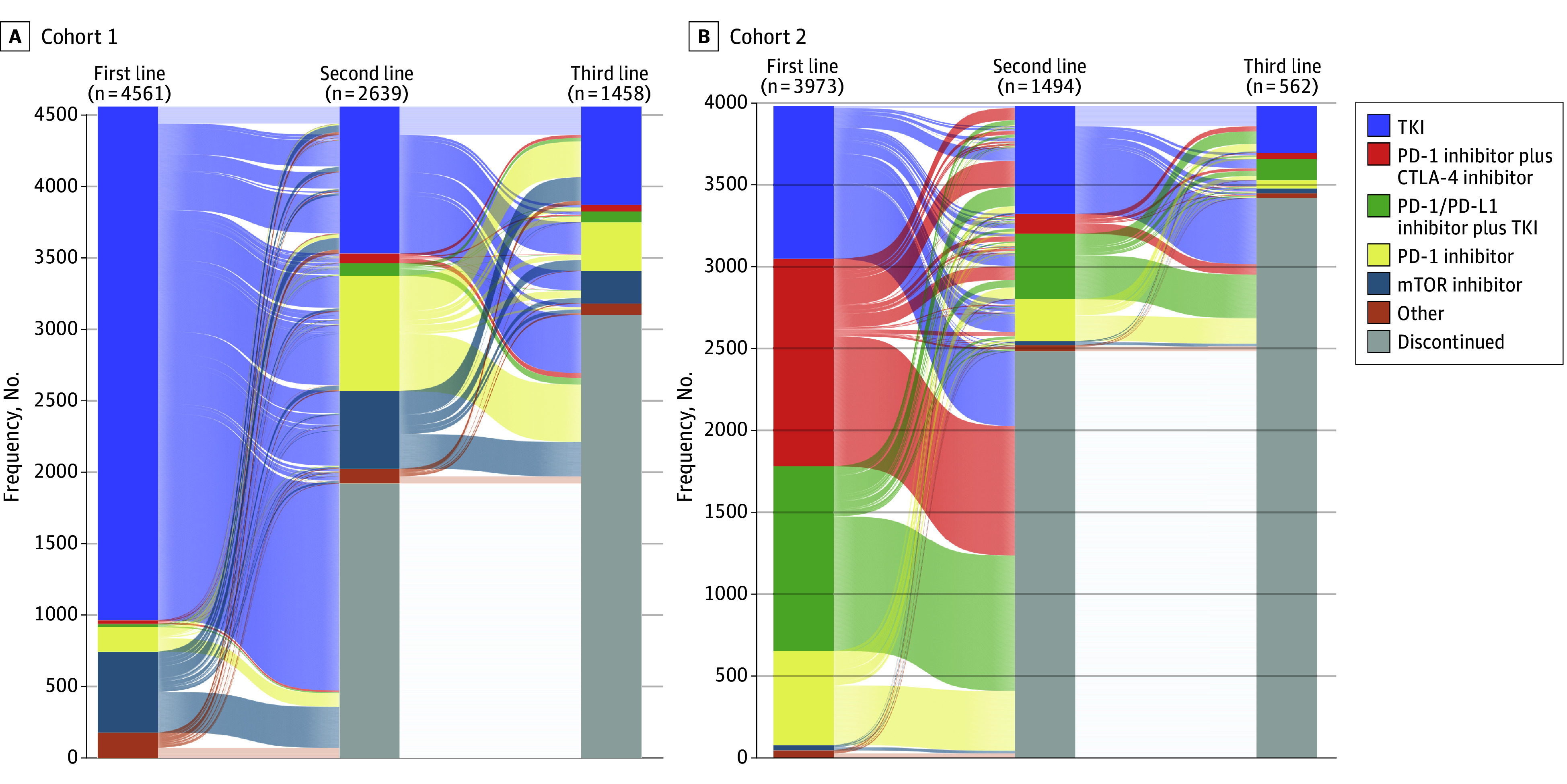
Treatment Trends Across Lines of Therapy in Patients With Metastatic Clear Cell Renal Cell Carcinoma A, Patients in cohort 1 (starting first-line therapy before April 16, 2018). B, Patients in cohort 2 (starting first-line therapy after April 16, 2018). CTLA-4 indicates cytotoxic T-lymphocyte–associated antigen 4; mTOR, mammalian target of rapamycin; PD-1, programmed cell death protein 1; PD-L1, programmed cell death ligand 1; TKI, tyrosine kinase inhibitor.

Among patients in the overall cohort, 4561 (53.4%) initiated first-line therapy before April 16, 2018 (ie, cohort 1), and 3973 (46.6%) initiated first-line therapy after April 16, 2018 (ie, cohort 2) (Figure 1). In cohort 1, 2639 patients (57.9%) and 1458 patients (31.9%) received second- and third-line therapies, respectively. Most patients in this cohort received TKI monotherapy across all lines of therapy. In cohort 2, 1494 patients (37.6%) received second-line therapy, and 562 (14.1%) received third-line therapy. Patients were more likely to receive first-line therapy with a dual ICI (1264 patients [31.8%]) or an ICI-TKI (1128 patients [28.4%]) combination regimen. Furthermore, TKI monotherapy was the most frequently used second-line (659 patients [44.1%]) and third-line (286 patients [50.9%]) regimen in this cohort (Table 1). Treatment trends across lines of therapy in both cohorts are summarized in Figure 2; eTable 2 in Supplement 1 summarizes the trends in different ICI-TKI combinations over time.

**Table 1.** Treatment Landscape of Patients With Metastatic Clear Cell Renal Cell Carcinoma Before and After Approval of Immune Checkpoint Inhibitor–Based Combinations

Treatment	Patients, No. (%)					
	First line		Second line		Third line	
	Cohort 1 (n = 4561)^a^	Cohort 2 (n = 3973)^b^	Cohort 1 (n = 2639)^a^	Cohort 2 (n = 1494)^b^	Cohort 1 (n = 1458)^a^	Cohort 2 (n = 562)^b^
TKI	3595 (78.8)	931 (23.4)	1029 (39.0)	659 (44.1)	688 (47.2)	286 (50.9)
PD-1 inhibitor plus CTLA-4 inhibitor	26 (0.6)	1264 (31.8)	69 (2.6)	119 (8.0)	48 (3.3)	39 (6.9)
PD-1/PD-L1 inhibitor plus TKI	23 (0.5)	1128 (28.4)	86 (3.3)	399 (26.7)	75 (5.1)	128 (22.8)
PD-1 inhibitor monotherapy	170 (3.7)	574 (14.4)	807 (30.6)	255 (17.1)	340 (23.3)	51 (9.1)
mTOR inhibitors	567 (12.4)	31 (0.8)	543 (20.5)	25 (1.7)	228 (15.6)	29 (5.2)
Other	180 (4)	45 (1.1)	105 (4)	37 (2.4)	79 (5.4)	29 (5.2)

Abbreviations: CTLA-4, cytotoxic T-lymphocyte–associated antigen 4; mTOR, mammalian target of rapamycin; PD-1, programmed cell death protein-1; PD-L1, programmed death ligand-1; TKI, tyrosine kinase inhibitor.

^a^ Before Food and Drug Administration approval of the first immune checkpoint inhibitor –based combination on April 16, 2018.

^b^ After Food and Drug Administration approval of the first immune checkpoint inhibitor –based combination on April 16, 2018.

### Treatment Landscape in the First-Line Setting

In the overall cohort, TKI monotherapy was the most commonly initiated first-line regimen (4526 patients [53.0%]), followed by ICI-based combinations (2441 patients [28.6%]) and ICI monotherapy with programmed cell death protein 1 (PD-1) inhibitors (744 patients [8.7%]). Patients in cohort 1 were more commonly treated with TKI monotherapy (3595 patients [78.8%]), followed by mammalian target of rapamycin (mTOR) inhibitors (567 patients [12.4%]) and PD-1 inhibitor monotherapy (170 patients [3.7%]) as their first-line treatment. Among patients in cohort 2, dual ICI and ICI-TKI combinations became the most common first-line treatment (2392 patients [60.2%]), followed by TKI monotherapy (931 patients [23.4%]) and PD-1 inhibitor monotherapy (574 patients [14.4%]).

### Treatment Landscape in the Second-Line Setting

TKI monotherapy (1688 patients [40.8%]) was the most commonly used second-line therapy across the overall cohort, followed by PD-1 inhibitor monotherapy (1062 patients [25.7%]) and ICI-based combinations (673 patients [16.3%]). In cohort 1, TKI monotherapy (1029 patients [39.0%]) was the most frequently used second-line regimen, followed by PD-1 inhibitor monotherapy (807 patients [30.6%]) and mTOR inhibitors (543 patients [20.6%]). In cohort 2, TKI monotherapy (659 patients [44.1%]) remained the most common, with ICI-TKI combination (399 patients [26.7%]) and PD-1 inhibitor monotherapy (255 patients [17.1%]) following.

### Treatment Landscape in the Third-Line Setting

**Figure 3.**
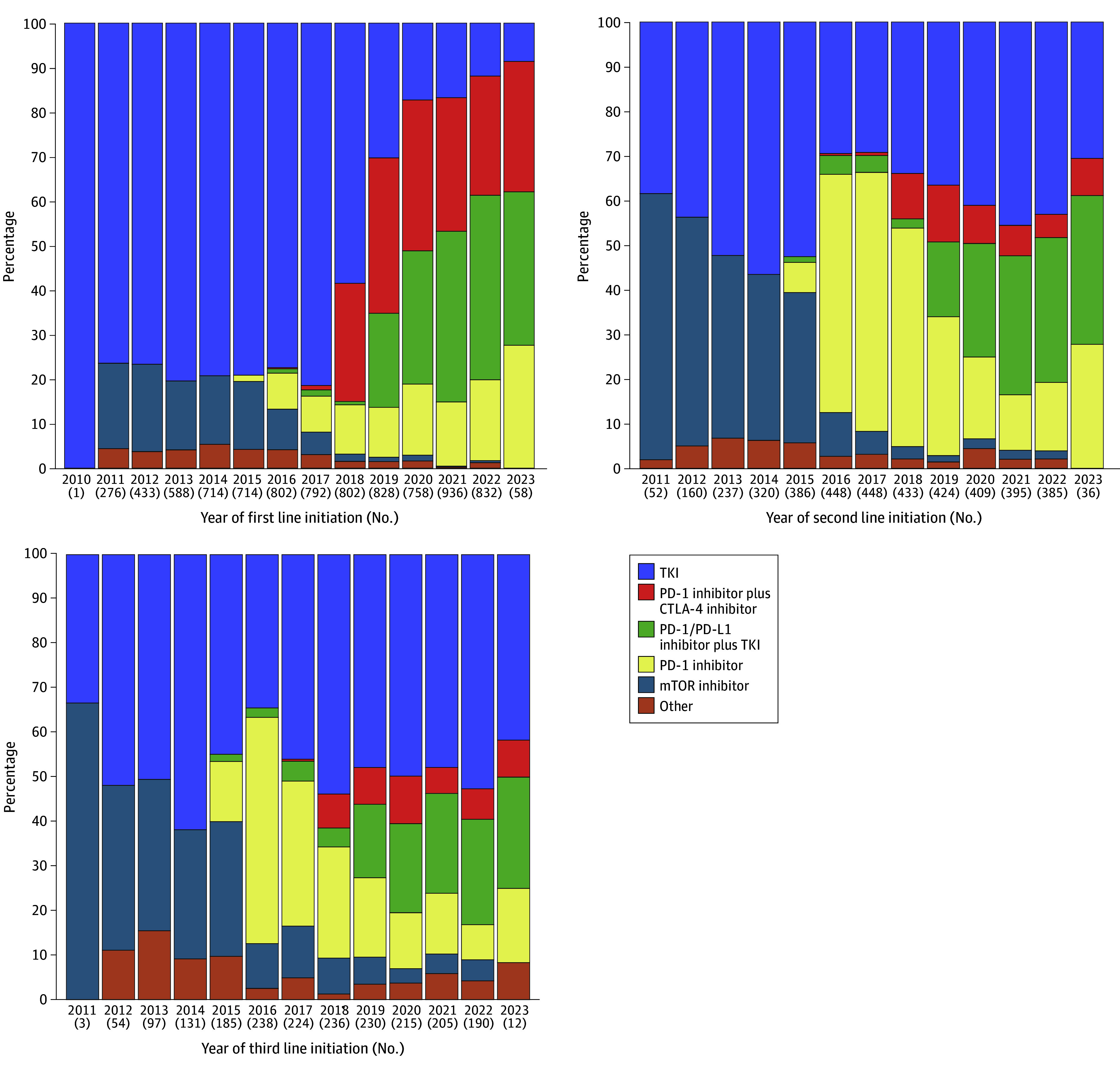
Treatment Trends by Line of Therapy in Patients With Metastatic Clear Cell Renal Cell Carcinoma CTLA-4 indicates cytotoxic T-lymphocyte–associated antigen 4; mTOR, mammalian target of rapamycin; PD-1, programmed cell death protein 1; PD-L1, programmed cell death ligand 1; TKI, tyrosine kinase inhibitor.

In the third-line setting, the most commonly used regimen was TKI monotherapy (974 patients [48.2%]) across the overall cohort, followed by PD-1 inhibitor monotherapy (391 patients [19.4%]), ICI-based combinations (290 patients [14.4%]), and mTOR inhibitors (257 patients [12.7%]). In cohort 1, TKI monotherapy (688 patients [47.2%]) was the predominant choice for third-line therapy, followed by PD-1 inhibitor monotherapy (340 patients [23.3%]) and mTOR inhibitors (228 patients [15.6%]). Cohort 2 showed a similar pattern, as most patients received TKI monotherapy (286 patients [50.9%]), followed by ICI-TKI combination (128 patients [22.8%]) and PD-1 inhibitor monotherapy (51 patients [9.1%]). Figure 3 displays the treatment trends across lines of therapy in the overall cohort, and eFigure 2 in Supplement 1 shows the trends in cohort 1 (eFigure 2A in the Supplement) and cohort 2 (eFigure 2B in the Supplement).

### Attrition Rate Analysis

At 1 year after discontinuing first-line therapy, most patients in cohort 1 received subsequent treatment (2622 patients [59.1%]), while 1382 patients (31.2%) died without further therapy, and 430 (9.7%) discontinued therapy and remained alive (Table 2). Similarly, in cohort 2, 1 year after discontinuing first-line therapy, most patients received second-line treatment (1120 patients [51.7%]), while 768 patients (35.4%) died within 1 year of treatment discontinuation, and 280 patients (12.9%) discontinued therapy and remained alive one year after treatment discontinuation. A similar trend was observed after second- and third-line therapy in both cohorts, with a majority of patients receiving subsequent lines of therapy.

**Table 2.** Attrition Rate Analysis at 1 Year and 6 Months After Treatment Discontinuation of Patients Before and After Approval of Immune Checkpoint Inhibitor–Based Combinations

Status at attrition rate analysis	Patients, No. (%)			
	Cohort 1^a^		Cohort 2^b^	
	1 y After treatment discontinuation	6 mo After treatment discontinuation	1 y After treatment discontinuation	6 mo After treatment discontinuation
**After first line**				
Received subsequent therapy	2622 (59.1)	2634 (59.0)	1120 (51.7)	1301 (52.1)
Died without subsequent therapy	1382 (31.2)	1287 (28.8)	768 (35.4)	799 (32.0)
Discontinued therapy/remained alive	430 (9.7)	543 (12.2)	280 (12.9)	397 (15.9)
Total	4434	4464	2168	2497
**After second line**				
Received subsequent therapy	1208 (56.8)	1210 (56.5)	630 (55.6)	727 (55.4)
Died without subsequent therapy	761 (35.7)	730 (34.1)	403 (35.6)	434 (33.1)
Discontinued therapy/remained alive	159 (7.5)	201 (9.4)	100 (8.8)	151 (11.5)
Total	2128	2141	1133	1312
**After third line**				
Received subsequent therapy	588 (59.6)	589 (59.6)	372 (55.9)	419 (54.6)
Died without subsequent therapy	332 (33.7)	314 (31.7)	244 (36.7)	282 (36.7)
Discontinued therapy/remained alive	66 (6.7)	86 (8.7)	49 (7.4)	67 (8.7)
Total	986	989	665	768

^a^ Before Food and Drug Administration approval of the first immune checkpoint inhibitor–based combination on April 16, 2018.

^b^ After Food and Drug Administration approval of the first immune checkpoint inhibitor–based combination on April 16, 2018.

## Discussion

To our knowledge, this is the largest study to assess treatment patterns and attrition rates in patients with metastatic ccRCC. We found that, before April 16, 2018, the first-line therapy for metastatic ccRCC predominantly consisted of TKI monotherapy, with only a small percentage of patients receiving mTOR inhibitors or PD-1/programmed cell death ligand-1 (PD-L1)-targeting therapies. However, following the approval of ICI-based combination therapies on April 16, 2018, the use of dual ICI and ICI-TKI regimens gradually increased in the first-line setting. Despite this trend, the adoption of these combination therapies remains suboptimal. Additionally, we observed an increased attrition in patients who began first-line treatment after April 16, 2018.

Our findings on the increasing use of dual ICI and ICI-TKI are consistent with the existing data. In a 2023 multicenter nationwide study, Shah et al^18^ documented an increased utilization of these regimens with a downtrend in the use of TKI monotherapy. Specifically, their study showed that the proportion of patients with metastatic ccRCC receiving TKI monotherapy decreased from 76.9% in the first quartile of 2018 to 26.9% at the end of 2020. In contrast, the use of the ipilimumab with nivolumab combination increased from 23.1% to 41.9% during the same period, and the use of the combination of pembrolizumab and axitinib rose from 7.7% in early 2019 to 31.3% by the end of 2020.^18^ The continued decrease in TKI monotherapy use in our study, down to 23.4%, is further encouraging. In 2022 and 2023, 19% of patients were receiving TKI alone, which may be explained by contraindications to ICIs, such as underlying autoimmune disease or history of organ transplantation. Notably, TKI monotherapy remains listed in the National Comprehensive Cancer Network (NCCN) guidelines as another recommended regimen for patients with metastatic ccRCC with any International Metastatic RCC Database Consortium (IMDC) risk disease. However, ICI-TKI combinations are listed as the preferred regimen regardless of IMDC risk. On the other hand, the dual ICI combination of ipilimumab and nivolumab is currently listed as a preferred regimen with a category 2A recommendation for patients with IMDC-favorable risk disease despite showing numerically better outcomes than sunitinib in this group in an 8-year update of the CheckMate 214 trial (median OS, 77.9 vs 66.7 months; hazard ratio [HR], 0.82 [95% CI, 0.60-1.13]).^16,23^

In phase 3 clinical trials, ICI-TKI combination regimens have demonstrated improved survival advantage compared with sunitinib in metastatic ccRCC, with a more pronounced benefit observed in patients with intermediate or poor IMDC risk compared with those with favorable risk.^11,12,24^ In a 2024 meta-analysis by Bolek et al,^25^ patients in the IMDC favorable risk group receiving ICI-TKI regimens had a 33% reduction in the risk of disease progression (HR, 0.67 [95% CI, 0.55-0.82]; *P* < .001) and a higher overall response rate (odds ratio [OR], 0.40 [95% CI, 0.28-0.57]; *P* < .001), but showed similar OS compared with sunitinib. These findings support the need for a more comprehensive predictive model to identify the subset of patients with favorable IMDC risk disease who would derive the most benefit from ICI-TKI regimens. Currently, these combinations should be preferred over TKI monotherapy for most patients, including those with favorable disease, while considering comorbidities and patient preferences.

In more advanced lines of therapy, our findings indicate an increased use of TKI monotherapy in the second and third-line settings for patients in cohort 2 compared with those in cohort 1. Current guidelines and recommendations list TKI monotherapy as the preferred subsequent therapy for patients with metastatic ccRCC, particularly for those with prior ICI exposure.^23^ Multiple retrospective analyses have shown that TKI monotherapy in patients with metastatic ccRCC, following progression on ICI or TKI monotherapy or ICI-TKI combinations, achieves an objective response rate (ORR) of approximately 22% to 41%, with a median progression-free survival (PFS) of 6.2 to 13.2 months.^26,27,28,29^ This suggests that TKI monotherapy remains effective after progression on ICI or TKI. Recent studies also show that cabozantinib demonstrates similar effectiveness as a second-line therapy, regardless of prior ICI-ICI or ICI-TKI regimens, supporting its use as a valid option after exposure to ICI-based combinations.^30^

Our findings also showed that a certain proportion of patients received subsequent ICI-based combinations after prior ICI exposure in the first-line setting. The efficacy of ICI rechallenge has recently been assessed in the CONTACT-03 trial, a randomized phase 3 study evaluating ICI-TKI vs TKI monotherapy in patients with prior progression on ICI-based regimen. This study failed to show any benefit of adding atezolizumab to cabozantinib (HR for death, 0.94 [95% CI, 0.70-1.27]; *P* = .69), with more adverse events in the combination group.^31^ Similar findings were validated in clinical settings.^32^ To evaluate whether this lack of benefit could be attributed to the use of a PD-L1 inhibitor and not a PD-1 inhibitor, the TiNivo-2 phase 3 trial assessed the addition of nivolumab to tivozanib (TKI) compared with tivozanib monotherapy and showed no improvement in outcomes (median PFS, 5.7 vs 7.4 months; HR, 1.10 [95% CI, 0.84-1.43]; *P* = .49).^33^ These data also support that ICI rechallenge should be discouraged in patients with metastatic ccRCC. Although our study demonstrated an increased use of ICI-TKI combination regimens in second-line therapy, our data cutoff (January 20, 2023) preceded the results of these trials. Future treatment sequencing trends in metastatic ccRCC should build upon these findings.

The treatment landscape of metastatic ccRCC will also evolve with the recent approval of belzutifan, a HIF-2α inhibitor, for patients who had progression on ICI and vascular endothelial growth factor (VEGF)-based therapies, based on LITESPARK-005 phase 3 trial, where belzutifan improved PFS compared with everolimus.^34,35^ Another oral agent targeting the HIF pathway is currently under investigation, demonstrating a promising safety profile with no reported cases of hypoxia (NCT04895748).^36^ Furthermore, the LITESPARK-012 (NCT04736706) phase 3 study is assessing the upfront combination of belzutifan with pembrolizumab and lenvatinib in the first-line metastatic ccRCC setting.^37^ Another pertinent question regarding treatment sequencing is the efficacy of first-line ICI-based therapies in patients who previously received 1 year of adjuvant pembrolizumab following definitive therapy, especially after this agent improved OS (HR for death, 0.62 [95% CI, 0.44-0.87]; *P* = .005).^38^ In this context, a 2024 report showed that, patients who received adjuvant ICI-based therapies achieved an ORR of 42% with both ICI-VEGF–targeting therapies and dual ICI regimens upon disease recurrence, confirming the efficacy of subsequent ICI-based treatments.^39^ Moreover, clinicians are actively seeking to expand the treatment landscape of RCC with new agents and novel mechanisms of action. The RadiCaL phase 2 study (NCT04071223) is evaluating radium-223 (radioligand) with cabozantinib in patients with metastatic ccRCC and bone lesions untreated with prior radiation therapy and no more than 2 prior lines of systemic therapy.^40^ The LITESPARK-024 phase 1/2 trial (NCT05468697) is investigating palbociclib (CDK4/6 inhibitor) with belzutifan in patients with advanced ccRCC and prior receipt of at least 2 systemic therapies.^41^

Regarding attrition rates, a recent clinical study in patients with metastatic ccRCC in the UK showed that among 1319 patients treated from January 2018 to the end of June 2021, only 59.2% received second-line treatment, and 23.5% received third-line therapy.^42^ Our study showed an increase in attrition rate in patients in cohort 2 compared with cohort 1. However, we observed that in cohort 2, the number of patients who discontinued therapy but were still alive 1 year after treatment discontinuation was numerically higher than in cohort 1. One plausible explanation could be the expected rate of complete responses with ICI-based combination therapies, which reached 12% with nivolumab plus ipilimumab in the CheckMate 214 trial,^16^ and ranged between 10% and 16% with ICI-TKI combinations.^11,43,44^ Additionally, this could be explained by a higher proportion of patients experiencing a treatment-free interval, given durable disease control.

### Strengths and Limitations

To our knowledge, this study is the largest to assess treatment patterns and attrition rates in 8534 patients with metastatic ccRCC over the course of 12 years and provides valuable data regarding the implementation of guidelines into clinical practice. The limitations of our study include the retrospective nature and data missingness in certain endpoints since it relies on EHR. Our dataset does not account for the association between treatment selection across lines of therapy and baseline characteristics, socioeconomic status, academic vs community setting, or patient’s comorbidities. Furthermore, patients lost to follow-up or receiving care outside the database’s coverage may introduce gaps in treatment information and attrition rates. Finally, our data cutoff of January 20, 2023, preceded the results of the CONTACT-03 and TiNivo-2 trials and the FDA approval of belzutifan, preventing us from assessing the impact of these developments on clinical practice.

## Conclusions

Our study showed increased utilization of ICI-ICI and ICI-TKI regimens as a first-line treatment for patients with metastatic ccRCC since their approval in April 2018. However, many patients still receive TKI or ICI monotherapy in this setting. Additionally, our findings suggest a continued high attrition rate after 2018. Considering that an important number of patients still die without receiving additional lines of therapy, ensuring appropriate first-line treatment in patients with metastatic ccRCC is of utmost importance. Upon external validation in larger cohorts, our results could guide treatment selection and patient counseling in the clinic.

## Notes

Supplement 1. **eTable 1.** Baseline Demographic Characteristics of Patients With Metastatic Clear Cell Renal Cell Carcinoma **eTable 2.** Number of Patients Receiving Approved ICI-TKI Combinations by Year **eFigure 1.** STROBE Flow Diagram **eFigure 2.** Treatment Trends by Line of Therapy in Patients With Metastatic Clear Cell Renal Cell Carcinoma

Supplement 2.
Data Sharing Statement
