# Supplementary material for: Treatment and Attrition Trends for Metastatic Clear Cell Renal Cell Carcinoma in the US
Source: JAMA Netw Open. 2025 Mar 20;8(3):e251201. doi: 10.1001/jamanetworkopen.2025.1201 (PMC11926653; doi:10.1001/jamanetworkopen.2025.1201)
Supplement: Supplement 1. — eTable 1. Baseline Demographic Characteristics of Patients With Metastatic Clear Cell Renal Cell Carcinoma eTable 2. Number of Patients Receiving Approved ICI-TKI Combinations by Year eFigure 1. STROBE Flow Diagram eFigure 2. Treatment Trends by Line of Therapy in Patients With Metastatic Clear Cell Renal Cell Carcinoma [file jamanetwopen-e251201-s001.pdf]

Supplemental Online Content

Ozay ZI, Jo Y, Galarza Fortuna G, et al. Treatment and attrition trends for metastatic clear cell renal cell carcinoma in the United States. *JAMA Netw Open.* 2025;8(3):e251201. doi:10.1001/jamanetworkopen.2025.1201

eTable 1. Baseline Demographic Characteristics of Patients With Metastatic Clear Cell Renal Cell Carcinoma

eTable 2. Number of Patients Receiving Approved ICI-TKI Combinations by Year

eFigure 1. STROBE Flow Diagram

eFigure 2. Treatment Trends by Line of Therapy in Patients With Metastatic Clear Cell Renal Cell Carcinoma

This supplemental material has been provided by the authors to give readers additional information about their work.

© 2025 Ozay ZI et al. *JAMA Network Open*.

eTable 1. Baseline demographic characteristics of patients with metastatic clear cell renal cell carcinoma

Variable	No. of patients (%) n = 8,534
Age, median (IQR), years	66 (59 – 74)
**Sex**	
Female	2,502 (29.3%)
Male	6,032 (70.7%)
**Race and ethnicity**	
Asian	131 (1.7%)
Black	629 (8.1%)
Hispanic or Latino	697 (9%)
White	5,493 (71%)
Missing	796
Other*	788 (10.2%)

*Other: non-Hispanic Alaska Native, American Indian, Native Hawaiian, or Pacific Islander or multiracial

Abbreviations: IQR: interquartile range.

© 2025 Ozay ZI et al. *JAMA Network Open*.

eTable 2. Number of patients receiving approved ICI-TKI combinations by year*

	Axitinib + pembrolizumab, n	Cabozantinib + nivolumab, n	Lenvatinib + pembrolizumab, n	Axitinib + avelumab, n
2017	-	12	-	-
2018	2	10	-	-
2019	233	18	1	11
2020	296	23	2	10
2021	286	160	56	6
2022	210	172	103	2
2023	15	10	8	1

*Patients receiving non-approved ICI-TKI combinations are not included in this table.

Abbreviations: ICI, immune checkpoint inhibitor; TKI, tyrosine kinase inhibitor.

© 2025 Ozay ZI et al. *JAMA Network Open*.

eFigure 1. STROBE Flow Diagram

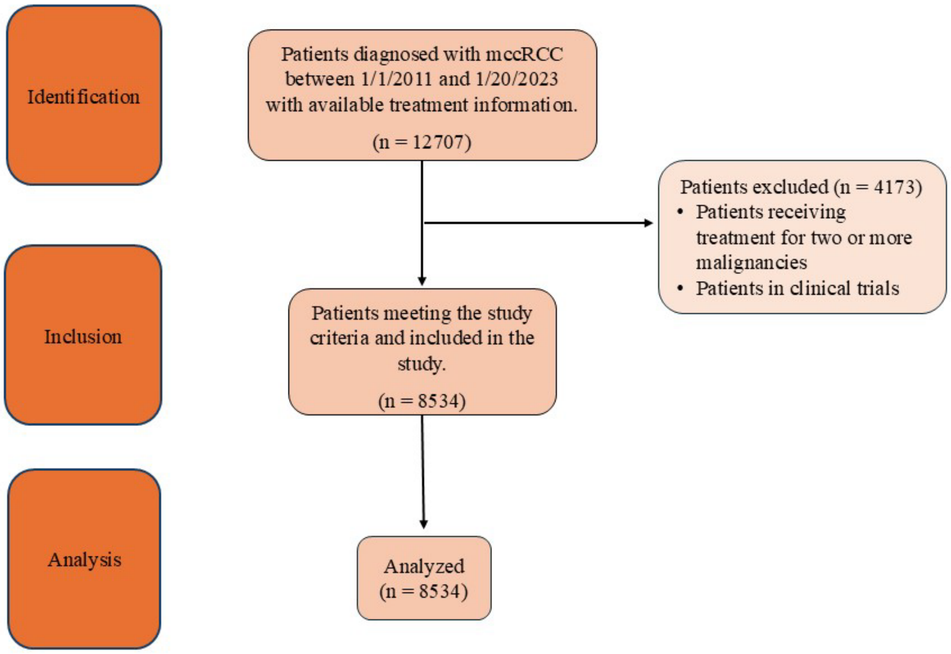

© 2025 Ozay ZI et al. *JAMA Network Open*.

eFigure 2.Treatment trends by line of therapy in patients with metastatic clear cell renal cell carcinoma

A) Patients in cohort 1 (initiating first-line therapy before April 16th, 2018)

B) Patients in cohort 2 (initiating first-line therapy after April 16th, 2018)

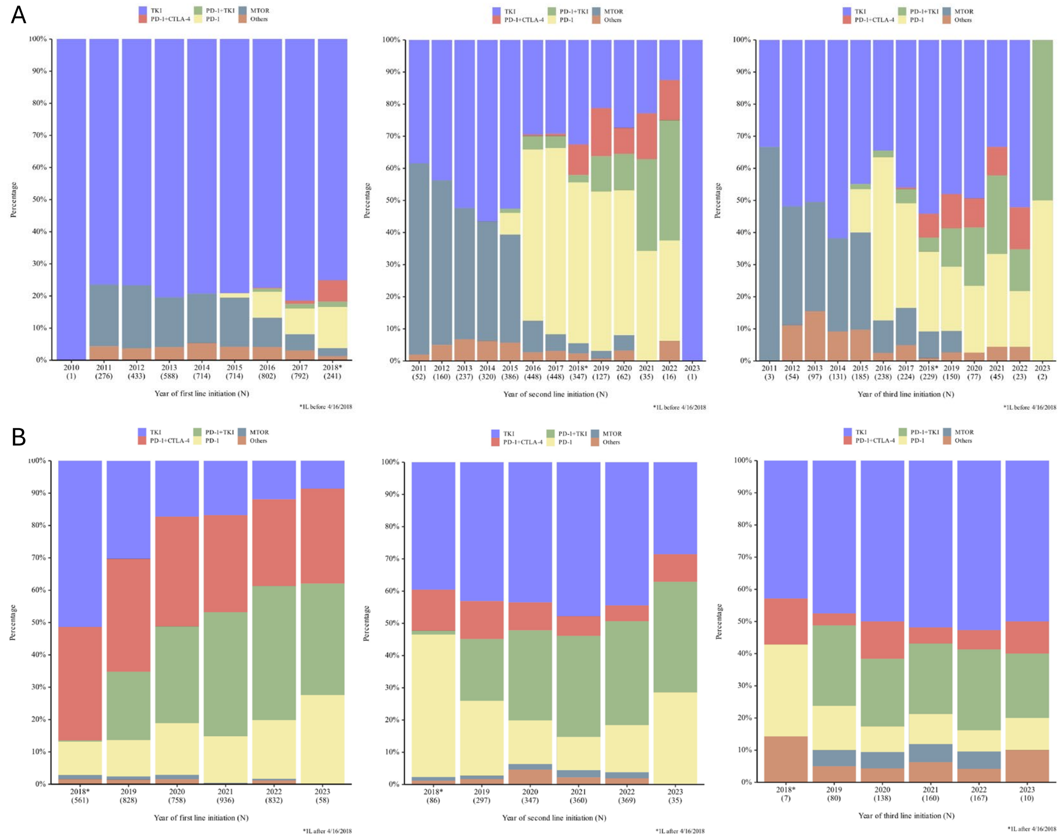

Abbreviations: CTLA-4, cytotoxic T-lymphocyte–associated antigen 4; mTOR, mammalian target of rapamycin; PD-1, programmed cell death protein 1; TKI, tyrosine kinase inhibitor.

© 2025 Ozay ZI et al. *JAMA Network Open*.
