# Supplementary material for: Treatment and Attrition Trends for Metastatic Clear Cell Renal Cell Carcinoma in the US
Source: JAMA Netw Open. 2025 Mar 20;8(3):e251201. doi: 10.1001/jamanetworkopen.2025.1201 (PMC11926653; doi:10.1001/jamanetworkopen.2025.1201)
Supplement: Supplement 2. — Data Sharing Statement [file jamanetwopen-e251201-s002.pdf]

Data Sharing Statement

Ozay. Treatment and Attrition Trends for Metastatic Clear Cell Renal Cell Carcinoma in the US. *JAMA Netw Open*. Published March 20, 2025. doi:10.1001/jamanetworkopen.2025.1201

Data
Data available: No

Additional Information
Explanation for why data not available: Data Sharing Statement Data available: The data that support the findings of this study have been originated by Flatiron Health, Inc. Requests for data sharing by license or by permission for the specific purpose of replicating results in this manuscript can be submitted to dataaccess@flatiron.com. Data types: De-identified participant data, Data dictionary How to access data: dataaccess@flatiron.com. When available: With publication Supporting Documents Document types: None Additional Information Who can access the data: Upon request and per consortium guidelines Types of analyses: Upon request and per consortium guidelines Mechanisms of data availability: Per consortium guidelines with data access agreement
